# Type of Renal Replacement Therapy (Hemodialysis versus Peritoneal Dialysis) Does Not Affect Cytokine Gene Expression or Clinical Parameters of Renal Transplant Candidates

**DOI:** 10.1155/2015/797490

**Published:** 2015-07-08

**Authors:** Dorota Kamińska, Katarzyna Kościelska-Kasprzak, Paweł Chudoba, Oktawia Mazanowska, Mirosław Banasik, Marcelina Żabinska, Maria Boratyńska, Agnieszka Lepiesza, Krzysztof Korta, Agnieszka Gomółkiewicz, Piotr Dzięgiel, Marian Klinger

**Affiliations:** ^1^Department of Nephrology and Transplantation Medicine, Wroclaw Medical University, Borowska 213, 50-556 Wrocław, Poland; ^2^Department of Vascular, General and Transplant Surgery, Wroclaw Medical University, Borowska 213, 50-556 Wrocław, Poland; ^3^Department of Histology and Embryology, Wroclaw Medical University, Chałubińskiego 6a, 50-368 Wrocław, Poland

## Abstract

Patients with renal failure suffer from immune disturbances, caused by uremic toxins and influenced by dialysis treatment. The aim of the present study was to reveal whether type of dialysis modality (hemodialysis, HD, versus peritoneal dialysis, PD) differentially affects the immunocompetence, particularly the expression of genes involved in the immune response. *Material*. 87 renal transplant candidates (66 HD, 21 PD) were included in the study. *Methods*. The peripheral blood RNA samples were obtained with the PAXgene Blood system just before transplantation. The gene expression of *CASP3, FAS, TP53, FOXP3, IFNG, IL2, IL6, IL8, IL10, IL17, IL18, LCN2, TGFB1*, and *TNF* was assessed with real-time PCR on custom-designed low density arrays (TaqMan). Gene expression data were analyzed in relation to pretransplant clinical parameters. *Results*. The mean expression of examined genes showed no significant differences between PD and HD with the exception of *FAS*, expression of which was 30% higher in PD patients compared to the HD group. There was nonsignificantly higher expression of proinflammatory cytokines in the PD group. The clinical inflammatory parameters (CRP, albumin, cholesterol, and hemoglobin levels) did not differ between the groups. *Conclusion*. Type of renal replacement therapy exerts no differential effect on cytokine gene expression or inflammatory clinical parameters.

## 1. Introduction

End stage renal disease (ESRD) is known to be a state of immune incompetence, which results in various immune abnormalities. Systemic and local microinflammation accompanied by immune deficiency is observed in patients with uremia. Inadequate responsiveness to vaccination, for example, hepatitis B, was described in patients with ESRD [1]. These patients are also more susceptible to viral as well as bacterial infections, which are the second (after cardiovascular) leading cause of mortality during renal replacement therapy [2]. The influence of malnutrition and inflammatory cytokines on cardiovascular risk, infection, and mortality rate has been extensively examined. Uremia and secondary hyperparathyroidism have been known since the 1990s to impact B and T lymphocyte function [3, 4] and antibody production [5].

Recently, uremia was reported to influence cytokine synthesis [6]. Partially this phenomenon can be explained by the neutrophil deficiency, as well as changes in neutrophil function in ESRD [7]. The chronic inflammation can be detected by elevated blood levels of C-reactive protein (CRP) and some cytokines, for example, IL-6, IL-18, IL-10, and TNF-alpha [8, 9]. Also abnormal function of regulatory T cells in uremia [10] and an imbalance of immature and memory B cells have been reported [11].

The above-described abnormalities are further enhanced by renal replacement therapy. Activation of the Th2 pathway with a potential tolerogenic effect was observed during hemodialysis [12]. Moreover, the peritoneal dialysis (especially with lactate-buffered peritoneal fluids) exerted deleterious effects on the Th1 cell subsets and promoted the Th2 type of immune response [13]. In contrast, some authors reported no influence of renal replacement therapy on cytokine and serum CRP levels [14].

While immune defects are well described in the literature, still it is not clear whether the type of dialysis modality (hemodialysis versus peritoneal dialysis) differentially affects the immunocompetence, particularly the expression of genes involved in the immune response and inflammation with the change in inflammatory gene profiles in patients on the active transplant waiting list. The aim of this study was to investigate pretransplant blood cytokine and apoptosis related gene expression to reveal possible difference in patients on hemodialysis (HD) and peritoneal dialysis (PD) in relation to clinical indices of malnutrition as well as inflammatory processes.

We focused on the immune factors known to be related to tissue injury and inflammation (IL-6, IL-8, IL-17, IL-18, NGAL, and TNF-alpha), apoptosis (Fas, caspase-3, and p53) and Th1 lymphocyte activation (IFN-gamma, IL-2), as well as Th2 (IL-10, TGF-beta1), and regulatory T cell function (FoxP3).

## 2. Material

The study was carried out on 87 renal transplant candidates (aged 16–72 years, mean 47 years; 34 females, 53 males) who had been admitted to hospital for transplantation surgery. Among them 66 patients were treated with hemodialysis and 21 patients with peritoneal dialysis. Patients' characteristics are presented in Table 1.

All HD patients were treated by low-flux hemodialysis on polysulfone membrane dialyzers with bicarbonate-containing solutions for 4 h three times weekly.

All patients from PD group were on continuous ambulatory peritoneal dialysis. The standard solutions of 2 l volume with various concentrations of glucose (mostly 3 times with 1.36/1.5%, and one time with 2.3%) were used. No icodextrin- or amino acid-containing solutions were used.

Weekly Kt/V > 1.7 was reported in all PD patients. In HD group in all subjects' single pool Kt/V per session was >1.2. Patients remained on renal replacement therapy from 1 to 97 months (mean 25 ± 18 months).

Blood samples for routine laboratory tests as well as gene expression were taken during immediate pretransplant examination before introducing immunosuppressive therapy. The samples were obtained at least 6 hours after the last HD session or at least 4 hours after the last peritoneal fluid removal.

Clinical data were obtained from medical records from patients' dialysis centers.

The project was performed after approval from the Commission of Bioethics at Wroclaw Medical University, and all aspects of the study were in accordance with the World Medical Association Declaration of Helsinki. Each patient read an information sheet and provided fully informed consent.

## 3. Methods

The peripheral blood samples were obtained with PAXgene Blood RNA tubes. RNA was isolated with a PAXgene Blood RNA kit (PreAnalytics) and reversely transcribed with a High Capacity RNA to cDNA kit (Applied Biosystems). The peripheral blood gene expression of caspase-3 (*CASP3*, Hs00263337_m1),* FAS* (*FAS*, Hs00236330_m1), p53 (*TP53*, Hs00153349_m1),* FOXP3* (*FOXP3*, Hs00203958_m1), IFN-gamma (*IFNG*, Hs00174143_m1), interleukin-2 (*IL2*, Hs00174114_m1), interleukin-6 (*IL6*, Hs00174131_m1), interleukin-8 (*IL8*, Hs00174103_m1), interleukin-10 (*IL10*, Hs00174086_m1), interleukin-17 (*IL17*A, Hs99999082_m1), interleukin-18 (*IL18*, Hs00155517_m1), NGAL (*LCN2*, Hs00194353_m1), TGF-beta (*TGFB1*, Hs99999918_m1), and TNF-alpha (*TNF*, Hs00174128_m1) was assessed with real-time PCR on custom-designed low density arrays (TaqMan) with TaqMan PCR Master Mix. All procedures were performed according to the manufacturer protocols.* GAPDH* (Hs99999905_m1) was chosen as a reference gene and proved to be invariant between the studied groups.

The expression data are presented as ΔΔCt = ΔCt_HD_ − ΔCt_sample_, where ΔCt_HD_ is the mean value of ΔCt_sample_ for HD group, ΔCt_sample_ = Ct_gene_ − Ct_*GAPDH*_, and Ct is the cycle threshold value and defines the calculated cycle number, in which the fluorescence measured during the PCR reaction increases over the preset threshold value. The above-mentioned equation provides expression data related both to the* GAPDH* reference gene and to the mean expression level for HD, which is considered a reference group here. The relative change in the observed expression of PD versus HD is calculated as 2^−ΔΔCt^ for mean ΔΔCt for PD.

### 3.1. Statistical Analysis

Statistical analysis was performed using the Statistica v.10 statistical package (StatSoft, Poland). The chi-square test was used to evaluate the difference in proportions between two groups. The assumption of normality was tested for each continuous variable. The difference between the two groups was evaluated with a *t*-test or, in the case of lack of normality, it was analyzed with the Mann-Whitney *U*-test. The correlations were tested with Pearson correlation or Spearman correlation, respectively. *p* < 0.05 was considered significant.

## 4. Results

### 4.1. Clinical Parameters

The groups of patients did not differ significantly according to age, gender, or BMI distribution. The time of renal replacement therapy was significantly shorter in the PD group compared to the HD group (median [IQR]: 15 [6–22] versus 24.5 [13–36] months, resp.).

The clinical pretransplant blood parameters are presented in Table 2. The clinical inflammatory parameters (CRP, albumin, cholesterol, and hemoglobin levels) and serum creatinine as well as iron level with transferrin saturation and iPTH did not differ between the groups. Only pretransplant serum uric acid concentration was significantly higher in the PD group (median [IQR]: 4.9 [4.1–5.7] versus 6.5 [5.8–8.0] mg/dL). Patient's age and CRP levels were lower in the PD group, but the differences did not reach statistical significance.

### 4.2. Gene Expression

The mean expression levels of the genes are presented in Table 3.* IL17* gene expression was undetectable in all patients.

**Figure 1:**
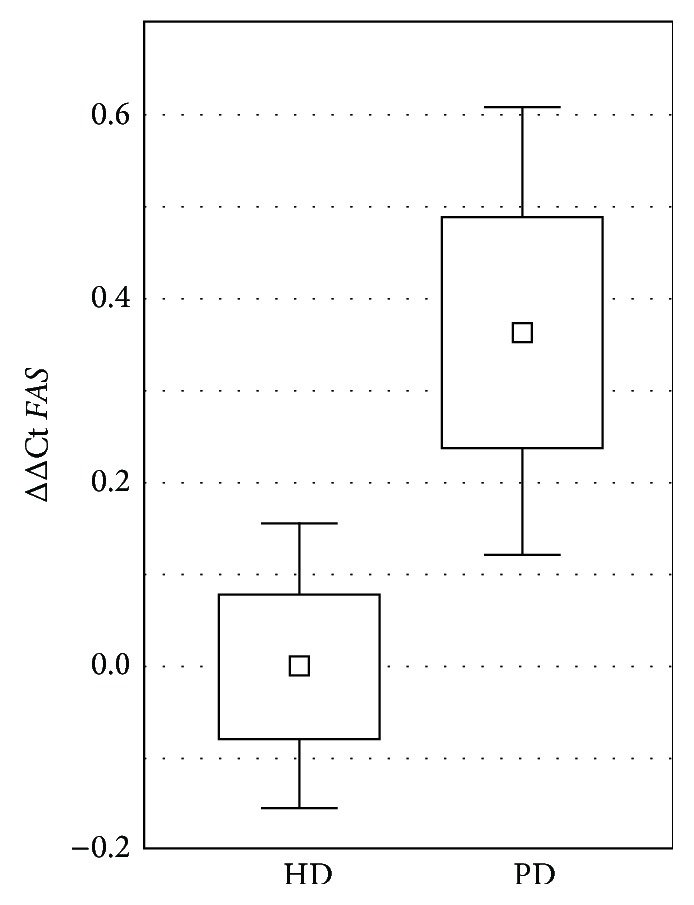
ΔΔCt describing relative gene expression of* FAS* versus* GAPDH* in peritoneal dialysis (PD) and hemodialysis (HD) group, related to the mean for HD samples. Graph presented as square: mean, box: mean ± SE, and whiskers: 95% CI.

The mean serum expression of the genes showed no significant differences between PD and HD with the exception of* FAS*, expression of which was 1.29 times higher (95% CI 1.03–1.61) in PD patients compared to the HD group (Figure 1).

Although higher gene expression of proinflammatory cytokines (*IFNG*,* IL6*, and* IL18*) in the PD group was noted, the differences were not statistically significant. Also, the lower* IL10* and higher* TGFB1* gene expression observed in the PD group were not statistically significant.

We found significantly lower (0.68x, 95% CI 0.50–0.93) relative expression of* IL10* to* FAS* in the PD group compared to the HD group (*t*-test, *p* = 0.013). We also observed lower (0.74x, 95% CI 0.55–1.01) relative expression of* IL10* to* IL18* of borderline significance (*t*-test, *p* = 0.054). Lower (0.60x, 95% CI 0.35–1.04) relative expression of* IL10* to* IFNG* in the PD group compared to the HD group did not reach statistical significance (*t*-test, *p* = 0.074).

The expression of some genes was related to clinical parameters of the patients. The correlations are presented in Table 4. The time of renal replacement therapy did not influence expression of any of the examined genes.

**Figure 2:**
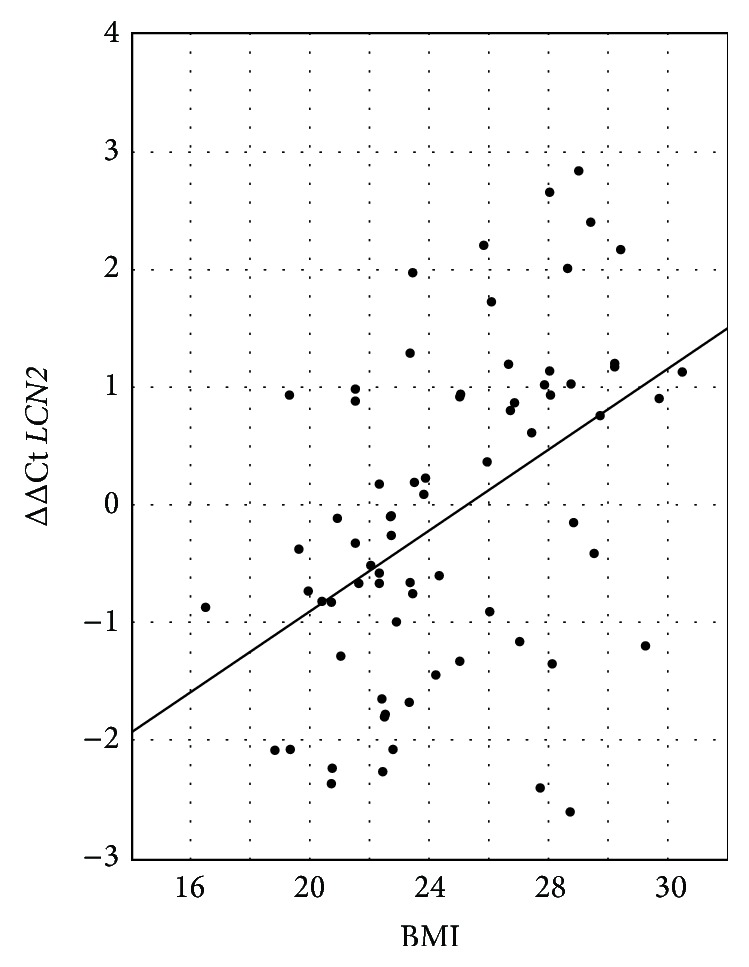
ΔΔCt describing relative gene expression of* LCN2* (NGAL) versus BMI.

*FOXP3* expression was negatively correlated with WBC in both groups (rs = −0.41, *p* = 0.01, and rs = −0.34), accompanied by* IL2* and* IL6* (rs = −0.33, *p* = 0.01, and rs = −0.34, *p* = 0.009, resp.).* IL10* gene expression correlated significantly with albumin level (rs = 0.41, *p* < 0.004).* LCN2* (NGAL) gene expression was positively correlated with WBC (rs = 0.44, *p* < 0.002). Also BMI was positively correlated with* LCN2* gene expression (rs = 0.44, *p* < 0.001) (Figure 2).

## 5. Discussion

End stage renal disease (ESRD) is associated with both inflammation and immune deficiency [11, 15, 16]. In our study we focused on the wide range of immune factors known to be related to inflammation, apoptosis, and lymphocyte Th1 and Th2 activation, as well as regulatory T cell function. It should be emphasized that the study encompassed the relatively healthy HD and PD patients just before transplantation. The general well-being of the patients allowed them to be qualified for renal transplantation and did not differ in terms of clinical indicators with the exception of the shorter dialysis treatment period in the PD group. In this selected group the main findings of our study were as follows: clinical symptoms of chronic inflammation in general did not differentiate the PD group from the HD group. Also the pro- and anti-inflammatory gene expression was similar in both examined groups. A uniform type of Th1 and Th2 activation pattern was seen in both groups.

Only an intensified apoptosis rate with increased* FAS* gene expression was seen in PD patients compared to HD patients. Lowered relative expression of* IL10* to proinflammatory cytokines (*IL18*,* IL10*,* IL6*, and* IFNG*) in the PD group compared to the HD group was also observed. The time of renal replacement therapy was the only clinical parameter that differentiated the HD group from the PD group. Nevertheless, the time of renal replacement therapy did not influence the expression of any of the examined genes. Therefore the observed upregulation of the* FAS* gene seems to be attributable solely to the type of dialysis.

Neutrophil deficiency and dysfunction due to not only uremic toxins but also malnutrition-inflammation-atherosclerosis (MIA) syndrome and dialysis was reported [7]. Chronic inflammation with acceleration of atheromatosis is responsible for the high rate of morbidity and mortality in dialysis patients [2]. T-helper lymphocytes were polarized towards the Th2 phenotype with higher synthesis of IL-4 and IL-10 in uremic patients and lower release of IFN-gamma [12]. Those reports suggest that uremia is associated with a predominance of Th2 cells and a switch of cytokine synthesis towards depressed cell-mediated immunity [17]. In our study we observed the universal pattern of immune activation in patients on the active waiting list with no switch to either a Th1 or Th2 response.

Overactivated but functionally compromised Tregs were described in ESRD. The basal* IL2* and* FOXP3* mRNA levels were high but did not rise upon stimulation [10]. An imbalance of Treg/Th17 function when compared to the healthy controls was described in the ESRD group [18, 19]. In our study the* IL17* gene expression was undetectable in all patients, so it was excluded from further analysis.

The laboratory indices of microinflammation include elevated blood levels of CRP and some cytokines, mainly IL-6, IL-18, IL-10, and TNF-alpha, which correlated with acceleration of atherosclerosis [8, 9, 20, 21]. Hypoalbuminemia was a risk factor for septicemia in dialysis patients [22]. Malnutrition may be caused by chronic inflammation via increased levels of proinflammatory cytokines [23]. No overt malnutrition was observed in our study patients. The study cohort consisted of patients on active waiting list, thus being a selected group of patients on RRT, healthier than average dialysis population. Also the detected albumin levels were within normal ranges for the majority of the patients. We observed that the albumin level correlated significantly with* IL10* gene expression in all patients and in the HD group. This is an expected finding because hypoalbuminemia was described as a potent factor augmenting chronic inflammation [24–26]. Anti-inflammatory mechanisms of IL-10 were reported to limit the production of a broad range of proinflammatory factors [27]. In our study we also observed that relative anti-inflammatory* IL10* gene expression to proinflammatory genes* IL6*,* IFNG*, and* IL18* was lower in the PD group compared to the HD group. This fact may indicate the intensified inflammatory process in the PD group.

Chronic activation of the immune system may be further intensified by renal replacement therapy. Several studies were performed to elucidate whether the type of the dialysis technique may influence the induction of a chronic inflammatory state.

The use of different peritoneal fluids was associated with disturbed Th1/Th2 balance. Use of lactate-buffered peritoneal fluid has deleterious effects on the Th1 cell when compared to both icodextrin solution and lactate/bicarbonate-buffered solution [13]. On the contrary no influence of biocompatibility of PD solutions on any parameter of MIA syndrome was reported [28]. Hwang et al. reported that* IL6* gene polymorphism influenced the peritoneal solute transport rate in PD patients [29].

Opposing reports of no influence of renal replacement therapy on cytokine and serum CRP levels have been published [14]. In our study the CRP level was negatively correlated with* TNF* gene expression only in the PD group. The major cellular origin of TNF-alpha is activated macrophages. The previous reports of the association between CRP levels and TNF-alpha expression were inconsistent, indicating that the presence of TNF-alpha in blood depends on a complex relationship in the inflammatory response and may not reflect biologic activity at the tissue level [21]. In our study, almost all the patients from the HD group were examined more than 6 hours after hemodialysis, so the effect of the single session of hemodialysis on gene expression was not taken into account.

Increased apoptosis of leukocytes associated with increased serum Fas and p53 protein levels as well as decreased antiapoptotic bcl-2 gene expression from neutrophils has been described in ESRD patients [30, 31]. In our study we found that in PD patients the expression of proapoptotic* FAS* was significantly higher than in HD patients. Moreover, the expression of proapoptotic* TP53* correlated negatively with WBC. The clinical meaning of this observation remains unclear, beyond a speculation on more balanced immune and inflammatory cell state in the PD transplant candidates. Also this observation supports the hypothesis that increased apoptosis of neutrophils influences total WBC in uremic patients.

Also we found that NGAL gene expression (*LCN2*) was positively correlated with WBC in all patients and the HD group. NGAL is a protein released mainly by neutrophils, but also renal tubular cells and some extrarenal cells, especially adipocytes [32, 33], and strongly depends on their absolute number, so the positive correlation with WBC is not surprising and confirms other reports [34]. Moreover, we found a strong correlation of NGAL gene expression with BMI, which is in agreement with other authors' reports [35] and supports the observation that NGAL is closely associated with obesity.

## 6. Limitations of the Study

Although according to our knowledge this is the first published report which compares cytokine and apoptosis-related gene expression directly between HD and PD patients in the pretransplant period, some potential limitations should be mentioned.

We analyzed data of more patients treated with HD than PD. The disparity between the number of patients treated with those two dialysis modalities reflects the global situation in renal replacement therapy. Less than 5% of patients with end stage renal disease are treated with PD in Poland which is similar to the world's situation when less than 11% of the patients are treated with PD.

This study was based on single time-point assessment of inflammatory cytokine expression and clinical coefficients. We had no possibility of assessing time-dependent changes in cytokine gene expressions. The concept was chosen on the basis that patients eligible for transplantation do not currently suffer from any acute infection. Usually they present a very good clinical status with high dialysis efficiency. All the patients at the time of transplantation were clinically stable, with no signs of inflammation, with no records of cancer, inflammatory diseases, or immunosuppressive therapy. Our results were obtained for a relatively homogeneous group of potential recipients on an active waiting list, who are not representative of all patients on renal replacement therapy.

## 7. Conclusion

Type of the renal replacement therapy exerts no differential effect on cytokine gene expression or inflammatory clinical parameters with the exception of an increased apoptosis rate with increased* FAS* gene expression in PD patients compared to HD patients. Also, related anti-inflammatory* IL10* gene expression towards proinflammatory cytokines was lowered in the PD group. Possibly uremia is the most potent factor which triggers immune activation, and further changes induced by the different types of renal replacement therapy are too subtle to be apparent. The intensity of the inflammatory response, measured by cytokine gene expression, may further influence the posttransplant alloimmune response and graft survival.

## Figures and Tables

**Table 1:** Subject characteristics.

	HD patients (*n* = 66)	PD patients (*n* = 21)	*p*
Age (years, mean ± SD, median [IQR])	48 ± 12 53.5 [19–72]	42 ± 14 40 [16–68]	0.084^*∗∗*^
Gender (female/male)	39 (59%)/27 (41%)	14 (67%)/7 (33%)	0.72
Time of dialysis (months, mean ± SD, median [IQR])	33 ± 42 24.5 [13–36]	15 ± 10 15 [6–22]	**0.017** ^**∗∗**^
BMI (kg/m^2^)	24.6 ± 3.6	24.8 ± 3.7	0.80^*∗*^
Primary disease			
Diabetic nephropathy	4 (6%)	5 (24%)	
Chronic glomerulonephritis	22 (33%)	12 (57%)	
Hypertensive nephropathy	7 (11%)	1 (5%)	
Polycystic renal disease	9 (14%)	1 (5%)	
Chronic interstitial nephritis	16 (24%)	0	
Unknown	8 (12%)	2 (9%)	

PD: continuous ambulatory peritoneal dialysis; ^*∗*^from *t*-tests; ^*∗∗*^from Mann-Whitney *U*-test.

**Table 2:** Clinical pretransplant laboratory parameters (mean ± SD, for not normally distributed values also median [IQR]).

	HD patients (*n* = 66)	PD patients (*n* = 21)	
Hgb (g/dL)	11.1 ± 1.7	10.8 ± 1.4	0.54^*∗*^
WBC count (×10^3^/mcL)	7.4 ± 2.3 7 [5.1–8.4]	7.5 ± 3.3 7.1 [5.8–8.8]	0.84^*∗∗*^
PLT count (×10^3^/mcL)	198 ± 64 225 [181–268]	230 ± 65 188 [155–237]	0.06^*∗∗*^
CRP (mg/L)	6.9 ± 7.0 1.2 [0.7–3.6]	3.2 ± 4.6 3.5 [1.2–6.6]	0.11^*∗∗*^
Cholesterol (mg/dL)	181 ± 57	206 ± 40	0.11^*∗*^
Albumin (g/dL)	4.2 ± 0.8	3.9 ± 0.8	0.31^*∗*^
Creatinine (mg/dL)	7.0 ± 2.0 5.6 [5.2–9.7]	7.4 ± 2.8 6.8 [5.1–8.6]	0.90^*∗∗*^
Uric acid (mg/dL)	5.4 ± 2.8 6.5 [5.8–8.0]	6.9 ± 1.4 4.9 [4.1–5.7]	0.015^**∗∗**^
iPTH (pg/mL)	320 ± 257 428 [221–797]	500 ± 371 256 [185–398]	0.29^*∗∗*^
Iron (mg/dL)	67.7 ± 27.2	69.1 ± 30.8	0.87^*∗*^
Transferrin saturation (%)	29.5 ± 11.1	26.2 ± 11.1	0.36^*∗*^

WBC: white blood cell; PLT: platelet; ^*∗*^from *t*-tests; ^*∗∗*^from Mann-Whitney *U*-test.

**Table 3:** Mean expression level of the studied genes in PD group presented as ΔΔCt in relation to mean value for HD. As a rule, the HD group mean value is always equal to zero.

Gene	PD (*n* = 21), mean ± SD
Apoptosis	
* CASP3 *	0.17 ± 0.97
*** FAS*** ^**∗**^	**0.36 ± 0.57**
* TP53 *	0.08 ± 0.42
Inflammation	
* IL6 *	0.17 ± 1.72
* IL8 *	0.15 ± 1.01
* IL18 *	0.19 ± 0.57
* TNF *	0.14 ± 0.45
* LCN2 *	0.05 ± 1.44
Th1	
* IFNG *	0.59 ± 1.54
* IL2 *	−0.19 ± 1.70
Th2	
* IL10 *	−0.26 ± 0.64
* TGFB1 *	0.15 ± 0.32
Treg	
* FOXP3 *	0.03 ± 0.70

^*∗*^ *t*-test *p* = 0.023.

**Table 4:** Statistically significant correlations between gene expression (ΔΔCt) and clinical parameters.

Clinical parameter	Gene	Correlation coefficient	*p*
BMI	*LCN2 *	0.44^*∗∗*^	<0.001

Age at KTx	*TP53 *	−0.32^*∗*^	0.028

WBC	*FOXP3 *	−0.41^*∗∗*^	0.001
	*IL2 *	−0.33^*∗*^	0.025
	*IL6 *	−0.34^*∗*^	0.020
	*LCN2 *	0.44^*∗*^	0.002
	*TNF *	−0.36^*∗∗*^	0.005
	*TP53 *	−0.26^*∗∗*^	0.043

Serum albumin level	*IL10 *	0.41^*∗*^	0.004

KTx: kidney transplantation; ^*∗*^Pearson correlation; ^*∗∗*^Spearman rank correlation.

## Abbreviations

BMI: Body mass index
*CASP3*: Caspase-3 gene
CRP: C-reactive protein
eGFR: Estimated glomerular filtration rate
*FAS*: First apoptotic signal gene
FoxP3: Forkhead box protein P3
*FOXP3*: Forkhead box protein P3 gene
*GAPDH*: Glyceraldehyde 3-phosphate dehydrogenase gene
HD: Hemodialysis
Hgb: Haemoglobin
IFN-gamma: Interferon gamma
*IFNG*: Interferon gamma gene
IL-2: Interleukin-2
*IL2*: Interleukin-2 gene
IL-6: Interleukin-6
*IL6*: Interleukin-6 gene
IL-8: Interleukin-8
*IL8*: Interleukin-8 gene
IL-10: Interleukin-10
*IL10*: Interleukin-10 gene
IL-17: Interleukin-17
*IL17*: Interleukin-17 gene
IL-18: Interleukin-18
*IL18*: Interleukin-18 gene
IQR: Interquartile range
*LCN2*: Neutrophil gelatinase-associated lipocalin gene
NGAL: Neutrophil gelatinase-associated lipocalin
p53: Tumor protein p53
PCR: Polymerase chain reaction
PD: Peritoneal dialysis
PLT: Platelet count
RRT: Renal replacement therapy
TGF-beta: Transforming growth factor beta
*TGFB1*: Transforming growth factor beta gene
TNF-alpha: Tumor necrosis factor alpha
*TNF*: Tumor necrosis factor alpha gene
*TP53*: Tumor protein p53 gene
WBC: White blood cell.
